# CX3CL1, a chemokine finely tuned to adhesion: critical roles of the stalk glycosylation and the membrane domain

**DOI:** 10.1242/bio.20149845

**Published:** 2014-11-13

**Authors:** Mariano A. Ostuni, Julie Guellec, Patricia Hermand, Pauline Durand, Christophe Combadière, Frédéric Pincet, Philippe Deterre

**Affiliations:** 1 INSERM, U 1135, Centre d'Immunologie et des Maladies Infectieuses, F-75013, Paris, France; 2 Sorbonne Universités, UPMC Université Paris 06, UMRS CR7, Centre d'Immunologie et des Maladies Infectieuses, F-75013, Paris, France; 3 CNRS, ERL 8255, Centre d'Immunologie et des Maladies Infectieuses, F-75013, Paris, France; 4 Sorbonne Universités, UPMC Université Paris 06, UMR 94550 ENS Laboratoire de Physique Statistique, F-75005, Paris, France; * Present address: INSERM, U 1134, Biologie Intégrée du Globule Rouge; Université Paris Diderot; Institut National de la Transfusion Sanguine, 6 rue Alexandre Cabanel, 75015, Paris, France.

**Keywords:** Chemokine, Adhesion, FRAP, Glycosylation, GPCR

## Abstract

The multi-domain CX3CL1 transmembrane chemokine triggers leukocyte adherence without rolling and migration by presenting its chemokine domain (CD) to its receptor CX3CR1. Through the combination of functional adhesion assays with structural analysis using FRAP, we investigated the functional role of the other domains of CX3CL1, i.e., its mucin stalk, transmembrane domain, and cytosolic domain. Our results indicate that the CX3CL1 molecular structure is finely adapted to capture CX3CR1 in circulating cells and that each domain has a specific purpose: the mucin stalk is stiffened by its high glycosylation to present the CD away from the membrane, the transmembrane domain generates the permanent aggregation of an adequate amount of monomers to guarantee adhesion and prevent rolling, and the cytosolic domain ensures adhesive robustness by interacting with the cytoskeleton. We propose a model in which quasi-immobile CX3CL1 bundles are organized to quickly generate adhesive patches with sufficiently high strength to capture CX3CR1+ leukocytes but with sufficiently low strength to allow their patrolling behavior.

## INTRODUCTION

The migration of circulating leukocytes to injury sites is an early step in the inflammation process and involves a sequence of coordinated interactions between leukocytes and endothelial cells (Luster et al., 2005; Langer and Chavakis, 2009). Chemokines, which are a family of low-molecular-weight soluble proteins that primarily attract leukocytes bearing the cognate receptors, are central to this physiological and pathological event (Charo and Ransohoff, 2006; Ransohoff, 2009). Chemokines trigger leukocyte activation and their firm adhesion to the inflamed endothelium, mainly through the mediation of integrins and their cognate ligands (Combadière et al., 2007; Simon et al., 2009; Speyer and Ward, 2011). Two of the members of the chemokine family are exceptions: CXCL16 and CX3CL1. In addition to their chemokine domain (called CD), these two chemokines possess three other domains: a mucin-like stalk, a transmembrane (TM) domain, and a cytosolic tail (Bazan et al., 1997; Matloubian et al., 2000). When interacting with their cognate receptors (CXCR6 and CX3CR1, respectively), these chemokines induce cell-cell adhesion (Ludwig and Weber, 2007). CXCL16 and CX3CL1 can also be cleaved by metalloproteinases, such as ADAM10 and ADAM17 (Garton et al., 2001; Hundhausen et al., 2003; Ludwig et al., 2005), to yield a soluble form that is chemotactic.

The CX3CL1 molecule, with its unique CX3CR1 receptor (Imai et al., 1997), is involved in adherence to the endothelium of the inflammatory monocyte population (CD14^hi^ CD16^−^ CX3CR1^+^ CCR2^+^ in humans, Ly6C^hi^ CX3CR1^+^ CCR2^+^ in mice) (Ancuta et al., 2003; Geissmann et al., 2003; Tacke and Randolph, 2006) likely through interaction with platelets (Schulz et al., 2007; Postea et al., 2012). This chemokine has also been implicated in the recruitment of NK lymphocytes (Guo et al., 2003; Lavergne et al., 2003) and in neuronal survival (Meucci et al., 2000; Mizuno et al., 2003; Cipriani et al., 2011; Kim et al., 2011). An additional intriguing function of the CX3CR1-CX3CL1 pair is to regulate the patrolling or “crawling” behavior of a minor monocytic subpopulation called “resident” or “non-classical” monocytes (CD14^lo^ CD16^−^+CX3CR1^+^ CCR2^−^ in humans, Ly6C^lo^ CX3CR1^+^ CCR2^−^ in mice) in blood vessels (Auffray et al., 2007). We recently showed that a monocytic subpopulation traffics and adheres to the bone marrow in a CX3CR1-CX3CL1 dependent manner (Jacquelin et al., 2013). For this patrolling behavior, the adhesion must be fast to capture monocytes, strong enough to allow sufficient time for screening the local environment, and small enough to screen as much territory as possible. This entails a precise adhesive potency, which is the subject of the present study, and we show that each domain of the CX3CL1 molecule plays a specific role in this adhesive behavior.

In the present paper, we show that CX3CL1, which is endowed with only one TM domain, has a lateral diffusion that is slower than that of the CX3CR1, which, similarly to all GPCRs, is a 7-TM molecule, and we demonstrate that this slow diffusivity is due to the glycosylation of the mucin stalk and the aggregation generated by the TM domain. We also found that both of these diffusion-limiting factors are highly required for the adhesiveness of the chemokine. Our data indicate that glycosylation ensures the accessibility of CX3CL1 to the CX3CR1 molecules buried in the membrane of the counter-adhesive cell. Moreover, we show that the only role of the CX3CL1 intracellular domain, which is involved in CX3CL1 recycling (Liu et al., 2005), is to increase the robustness of the adhesion. Our data allow the derivation of a model in which the formation of adhesive patches is controlled by the dynamics of the CX3CR1 receptors that bind the CD domain presented by a defined number of ligands prearranged in quasi-immobile bundles. This model is consistent with the observed patrolling behavior of CX3CR1-positive leukocytes.

## RESULTS

### The intracellular domain and the mucin stalk of CX3CL1 are required for a strong adhesive potency

**Fig. 1.**
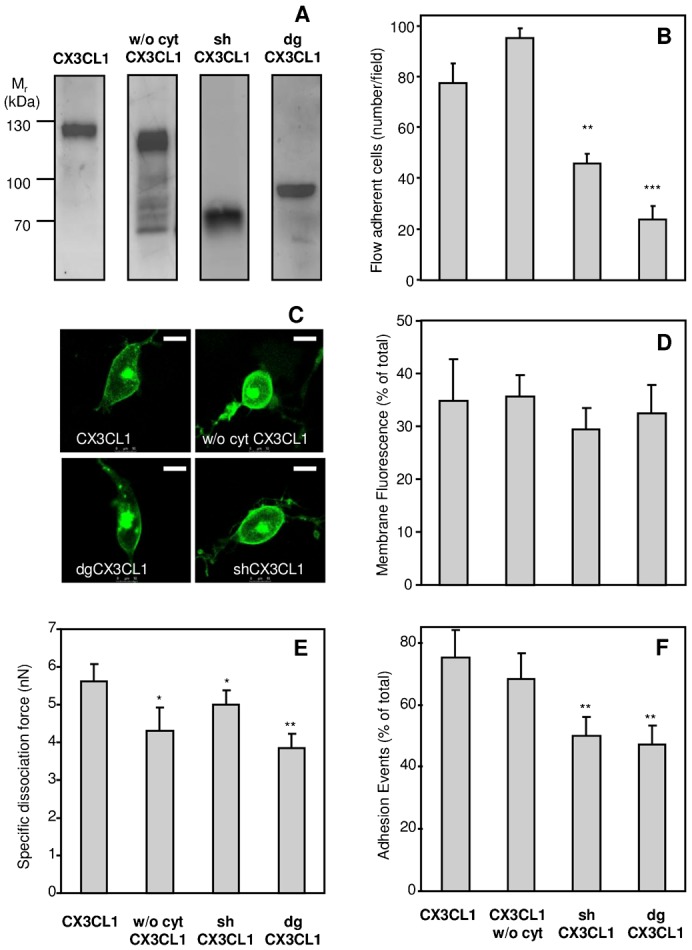
Adhesive potency of the truncated and deglycosylated CX3CL1 mutants compared to native CX3CL1. (A) Immunoblotting of the different CX3CL1-EYFP chimeras expressed in the COS7 cell line with anti-CX3CL1 mAb. Thirty micrograms of membrane lysates from COS7 cells expressing CX3CL1 chimeras were loaded on a 10% polyacrylamide gel under reducing conditions, transferred to a nitrocellulose membrane, and incubated overnight at 4°C under agitation with anti-human CX3CL1 primary antibody. The immune complexes were visualized with secondary peroxidase-conjugated antibodies using a chemiluminescent kit. (B) Number of CX3CR1+ CHO cells adhering to a monolayer of COS7 cells expressing various forms of CX3CL1-EYFP chimera. The results were obtained using a flow adherence assay. ** p<0.01 and *** p<0.001 compared with native CX3CL1-EYFP. (C) Membrane localization of CX3CL1 and its variants. Representative images of COS7 cells expressing EYFP chimeras of CX3CL1, CX3CL1 w/o cyt, shCX3CL1, or dgCX3CL1. The images were taken with a Leica SP5 confocal microscope (63× dry objective). The bars represent 10 µm. (D) The membrane expression of the various constructs was evaluated by confocal microscopy as a ratio of membrane to total fluorescence. (E) Dissociation force, assessed by the two pipette assay, between CX3CR1+ CHO-cells and COS7 cells expressing the indicated CX3CL1-EYFP chimera. (F) Percentage of positive adherent cell doublets calculated for each CX3CL1-EYFP chimera. * p<0.05 and ** p<0.01 compared with native CX3CL1-EYFP.

We first tested the contribution of different features of the CX3CL1 molecule to its adhesiveness, i.e., the presence of its cytosolic tail, the length of its mucin stalk, and its glycosylation. To this end, we constructed three CX3CL1 mutated analogues: one without the cytosolic tail (termed “w/o cyto CX3CL1”), one with half of the mucin stalk (termed “shCX3CL1”), and one with a fully deglycosylated stalk (termed “dgCX3CL1”), which was obtained by mutating the 52 serine or threonine residues of the mucin stalk to alanine, i.e., all of the potential sites for O-glycosylation. To follow the cellular expression of CX3CL1, these molecules were linked to EYFP at the C terminus, whose presence was indeed previously shown to not impair the function of the whole protein (Liu et al., 2005; Hermand et al., 2008; Huang et al., 2009). After expression in the COS7 cell line, the molecular weight of the CX3CL1-EYFP chimera leveled at 120 kDa, i.e., the sum of the complete CX3CL1 (90 kDa) and of the EYFP protein (30 kDa). Consistently, the w/o cyto CX3CL1 exhibited a molecular weight of 110 kDa, whereas the shCX3CL1 mutant had a weight of approximately 70 kDa (i.e., the sum of the 40 kDa short CX3CL1 plus the EYFP protein). The weight of the dgCX3CL1 mutant was 80 kDa, i.e., the sum of the polypeptide of CX3CL1 (50 kDa) plus the EYFP protein (Fig. 1A). The existence of some minor bands of lower molecular weight as seen in the w/o cyto CX3CL1 well is probably due to some intracellular forms of CX3CL1 that are not well processed as previously analyzed in detail (Garton et al., 2001).

The adhesive potency of these mutated proteins was first tested using the classical flow chamber assay (Fong et al., 1998; Haskell et al., 2000; Daoudi et al., 2004; Hermand et al., 2008), in which CX3CR1-positive CHO cells (CX3CR1+) were circulated at 1.5 dyne/cm^2^ over adherent COS7 cells expressing the CX3CL1-EYFP chimera. The number of CX3CR1+ cells that adhered to COS7 cells expressing the w/o cyto CX3CL1 was found to be unchanged (if not higher) compared to those adhering to the COS7 cells expressing the CX3CL1 native molecule (Fig. 1B). In contrast, the number of CX3CR1+ cells adhering to COS7 cells expressing shCX3CL1 or dgCX3CL1 was 2-fold and 4-fold lower, respectively (Fig. 1B). Moreover, we used confocal microscopy to confirm that the three CX3CL1 variants were well expressed at the COS7 external membrane (Fig. 1C) and that the proportion of the membrane-targeted proteins to the whole expression was similar for the four constructs (Fig. 1D).

The adhesive potency of the CX3CL1 variants was also tested through a dual pipette assay giving access to the force required to dissociate a cell doublet (Daoudi et al., 2004). We found that cells expressing the w/o cyto CX3CL1 displayed only a 4-nN dissociation force toward cells expressing the CX3CR1 receptor, whereas the native molecule developed a 5-nN force (Fig. 1E) for a similar number of positive adhesive events (Fig. 1F). The dissociation force of both the shCX3CL1 and dgCX3CL1 variants was also significantly reduced (Fig. 1E), and the probability of forming adhesive doublets was 40% decreased compared with the native CX3CL1 (Fig. 1F). We previously showed using a BRET assay that the native CX3CL1 molecule forms aggregates (Hermand et al., 2008): we then verified here that dgCX3CL1 showed a BRET at an amplitude similar that of native CX3CL1 (supplementary material Fig. S1).

Taken together, these data show that the shortening of the mucin stalk of CX3CL1 and the mutation of the potentially glycosylated residues of the mucin stalk are both deleterious for the adhesive potency of the chemokine to a larger extent than the absence of the cytosolic domain. To understand at the molecular level the reasons underlying this decreased adhesion, we tested three possible causes: lesser accessibility of the CD, change in the supramolecular organization via the cytoskeleton, and impairment of CX3CL1 lateral diffusion.

### The mucin stalk is maintained extended by glycosylation to present the CD to the receptor

**Fig. 2.**
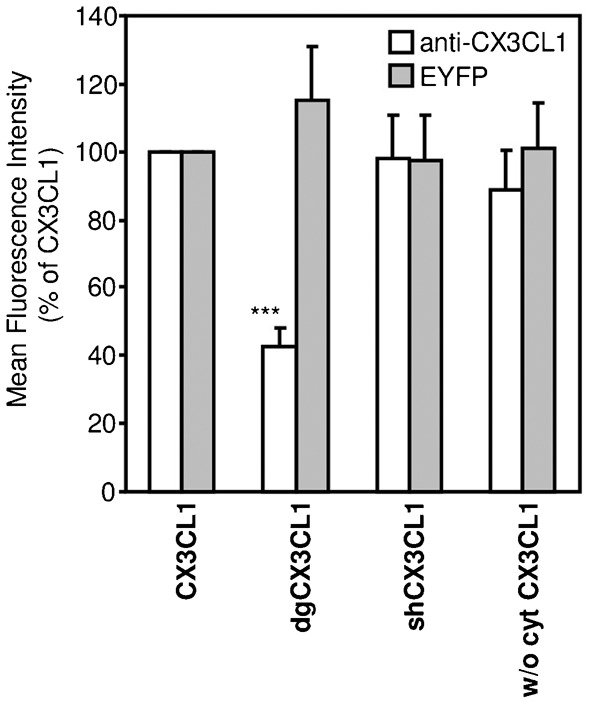
Analysis of the truncated and deglycosylated CX3CL1-EYFP chimera by immunostaining. Flow cytometry signal from different CX3CL1-EYFP chimera revealed by EYFP fluorescence (filled bars) or by anti-CX3CL1 PE-conjugated antibody (empty bars). *** p<0.001 compared with native CX3CL1-EYFP.

COS7 cells transiently transfected with the three different CX3CL1-EYFP chimeras and labeled with anti-CX3CL1-PE antibody were analyzed by FACS. All of the chimera constructions gave similar EYFP signals (Fig. 2, grey bars) and were similarly targeted at the external membrane (Fig. 1C,D). However, dgCX3CL1 showed a marked decrease in staining by the anti-CX3CL1-PE antibody (Fig. 2, empty bars), indicating that the antibody accessibility was lowered. These results, together with the flow adhesion results, strongly suggest that the accessibility of the CD to both antibody (Fig. 2) and CX3CR1 (Figs 1 and 2) is compromised when the chemokine is deglycosylated.

The adhesion and FACS measurements (Figs 1 and 2) with shCX3CL1 also support this interpretation. A shorter mucin stalk presents the CD outside the membrane in such a way that it is accessible to a free molecule in solution, such as an antibody (Fig. 2). However, because it remains closer to the membrane, it is less likely to encounter a transmembrane protein from another cell, such as CX3CR1. This reasoning explains why the probability of adhesion (flow chamber and dual pipette assay) is reduced (Fig. 1B). The decrease in adhesion strength (Fig. 1E) obtained in the dual pipette assay was also likely due to a lesser number of CX3CR1-CX3CL1 bonds.

### The cytosolic domain ensures the robustness of the adhesion via the cytoskeleton

The data obtained with the w/o cyto CX3CL1 mutant using the dual pipette assay suggest that the CX3CL1 molecule without its cytosolic domain has the same probability to functionally bind the CX3CR1 receptor in the counter-adhesive cell as the native CX3CL1 (Fig. 1F), but that its resistance to radial force is slightly less, likely due to some impaired link to the cytoskeleton (Fig. 1E). Among the components of the cytoskeleton, we specifically explored the potential role of the actin network. We found that treatment with latrunculin B, which inhibits actin polymerization (Wakatsuki et al., 2001), leads to a 25% decrease in the dissociation force (supplementary material Fig. S2), i.e., the level obtained in the absence of the cytosolic tail. Thus, it is conceivable that a connection between CX3CL1 and the actin cytoskeleton is required to ensure robust adhesion. However, this potential connection appears dispensable to the capture of adhering cells in the flow assay (Fig. 1B) likely because the force displayed by w/o cyto CX3CL1 is sufficient to resist the flow rate used in our assay.

### The lateral diffusion of CX3CL1 is slower than that of CX3CR1

Since the lateral diffusion of an adhesion molecule is crucial for its adhesive function (Dustin and Springer, 1991; Kucik et al., 1996; Kusumi et al., 1999; Kucik et al., 2001; Yu et al., 2010), we investigated the membrane diffusion of CX3CL1 and CX3CR1 through the FRAP technique.

**Fig. 3.**
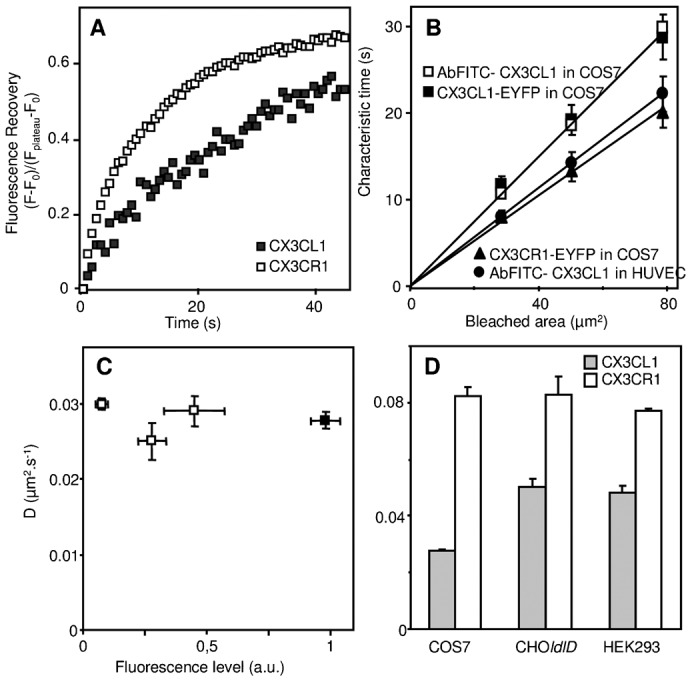
Analysis of the lateral diffusion of CX3CL1 and CX3CR1 in various cell types. (A) Kinetics of the fluorescence recovery after photobleaching of a 5-µm-diameter bleached area at the surface of COS7 cells expressing CX3CL1-EYFP (filled squares) or CX3CR1-EYFP (empty squares) chimeras. (B) Dependence as a function of the surface of the bleached area of the FRAP characteristic time of CX3CL1 expressed in COS7 cells either as an EYFP chimera (filled squares) or bound to FITC-stained specific antibody (empty squares), of FITC-Ab bound CX3CL1 in HUVEC cells (filled circles), and of CX3CR1-EYFP expressed in COS cells (filled triangles). (C) The diffusion rate of CX3CL1-EYFP was measured at the surface of COS7 cells expressing different CX3CL1-EYFP levels as the membrane fluorescence level measured by confocal microscopy. The black square represents the data obtained using COS7 transfected under the conditions that were classically used throughout this study. (D) The diffusion rates of CX3CL1-EYFP (filled bars) and of CX3CR1-EYFP (empty bars) were measured in different cell lines as indicated.

We first transiently transfected the EYFP chimera into COS7 cells and found that the mobile fraction of both CX3CL1 and CX3CR1 represented approximately half of the whole membrane population (Tables 1 and 2) and that the time of recovery after bleaching of a 5-µm-diameter spot was clearly longer for the mobile fraction of CX3CL1 (Fig. 3A, full squares, t_1/2_ = 31.5±2.79 s) than for CX3CR1 (Fig. 3A, empty squares, t_1/2_ = 13.5±0.73 s). This recovery was most likely due to actual lateral diffusion because we found that the characteristic time of diffusion was directly proportional to the surface of the bleached area (Fig. 3B), confirming that the fluorescence recovery was not due to the potential arrival of cytosolic CX3CL1 in the bleached membrane area. In addition, this experiment showed that, at least in the distance range of 3–5 µm, CX3CL1 and CX3CR1 displayed Brownian motion without constraints, i.e., neither “microdomains” nor “fences” (He and Marguet, 2008). We further ensured that the slow diffusion of the CX3CL1-EYFP chimera is not due to the overexpression of the protein (Fig. 3C): the same diffusion rate was found with a CX3CL1 expression level that was 2- to 10-fold (Fig. 3C, empty squares) lower than that currently obtained (Fig. 3C, filled square).

**Table 1.**
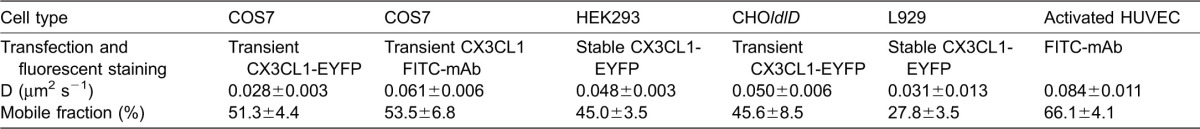
Characteristics of the lateral diffusion rate of CX3CL1 in various cell types detected using EYFP chimera or antibody staining

**Table 2.**
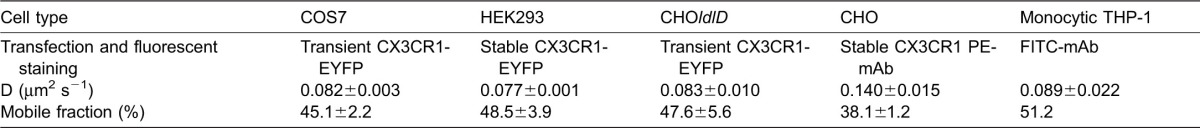
Characteristics of the lateral diffusion rate of CX3CR1 in various cell types and detected using EYFP chimera or antibody staining

The data obtained with the COS7 cell line indicate that the diffusion rate of CX3CL1 is approximately 3×10^−2^ µm^2^ s^−1^ (Fig. 3D, grey bars), whereas that of CX3CR1 in the same cell type is approximately 8×10^−2^ µm^2^ s^−1^ (Fig. 3D, empty bars). We then assessed whether the low rate of CX3CL1 lateral diffusion is also found in other cell types. In CHO*ldlD*, HEK293, and L929 cells (murine fibroblastic origin) (Fig. 3D; Table 1), the diffusion rate of the CX3CL1-EYFP chimera in the cell surface was between 3 and 5×10^−2^ µm^2^ s^−1^, i.e., within a similar range as in COS7 cells. Furthermore, we verified that the bleaching mode had no influence on the diffusion rate data. Using the mild bleaching mode with a fringe pattern (Fluorescence Recovery After Photobleaching Pattern, FRAPP) (Davoust et al., 1982; Binks et al., 1989; Gambin et al., 2006), we found that CX3CL1 and CX3CR1 displayed a diffusion rate in HEK293 cells similar to that obtained using classical FRAP (supplementary material Table S1). We also confirmed that this diffusion was indeed lateral diffusion because the same results were obtained when the fluorescence is monitored in the TIRF mode (supplementary material Table S1). The analysis of the data obtained using this technique showed that the fluorescence recovery of CX3CL1 was purely exponential, indicating that the diffusive objects are essentially monodisperse in size (supplementary material Fig. S3).

We then used FRAP to analyze the diffusion of CX3CL1 in primary cells. To this end, we stained the chemokine with fluorescent antibodies and found that the diffusion of CX3CL1 in activated primary HUVEC cells was similar to that obtained with COS7 cells transfected with CX3CL1 (Table 1). Furthermore, CX3CL1 expressed by activated HUVEC cells also diffused in a Brownian mode (Fig. 3B). The chemokine stained with mAb was surprisingly found to be faster (8×10^−2^ µm^2^ s^−1^) than the EYFP-chimera (Table 1). This is probably due to some slight restriction of lateral diffusion due to the addition of the intracellular EYFP moiety, which could slow down the whole molecule by interacting with some intracellular component. Because CX3CR1 is primarily expressed in circulating monocytes, we measured its diffusion rate in the monocytic cell line THP-1 and found a diffusion rate of approximately 9×10^−2^ µm^2^ s^−1^ (Table 2). We concluded that the CX3CR1, which, as a GPCR, possesses 7 TM domains and is likely found as a dimer (Darbandi-Tehrani et al., 2010), is always more diffusive than its ligand CX3CL1, which is endowed with only one TM domain.

### Glycosylation of the mucin stalk is responsible for the slow CX3CL1 diffusivity

**Fig. 4.**
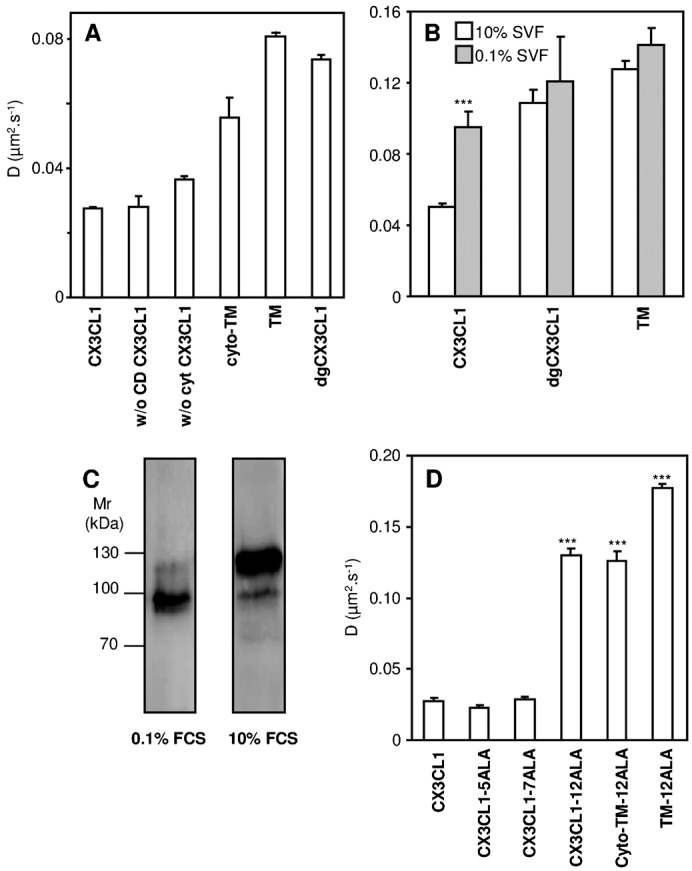
Lateral diffusion rate of CX3CL1-EYFP and its truncated, deglycosylated, or TM mutants. (A) Diffusion rate assessed by FRAP in COS7 cells of native CX3CL1-EYFP or CX3CL1-EYFP lacking its chemokine domain (named “w/o CD CX3CL1”), its cytosolic tail (named “w/o cyto CX3CL1”), its extracellular part (named “cyto-TM”), or both of its extracellular and intracellular regions (named “TM”) or deglycosylated (named “dgCX3CL1”); * p<0.05 and *** p<0.001 compared with native CX3CL1-EYFP. (B) Diffusion rate of CX3CL1-EYFP and various mutants as assessed by FRAP in CHO*ldlD* cells cultured either with 10% FCS (empty bars) or 0.1% FCS (filled bars) medium. *** p<0.001 compared with the respective 10% FCS cultured CX3CL1-EYFP-expressing cells. (C) Immunoblotting of CX3CL1 chimera in CHO*ldlD*. Thirty micrograms of membrane lysates from CHO*ldlD* transfected with CX3CL1-EYFP and cultivated under normal (right) or deprived (left) serum conditions were loaded onto a 10% polyacrylamide gel under reducing conditions, transferred to a nitrocellulose membrane, and incubated overnight at 4°C under agitation with anti-human CX3CL1 primary antibody. The immune complexes were visualized with secondary peroxidase-conjugated antibodies using a chemiluminescent kit. (D) Lateral diffusion measured by FRAP in COS7 cells expressing the native CX3CL1-EYFP chimera or different constructions in which residues 321 to 325 were mutated to ALA (named “CX3CL1-5ALA”), residues 326 to 332 were mutated to ALA (“CX3CL1-7ALA”), or residues 321 to 332 were mutated to ALA (“CX3CL1-12ALA”). The lateral diffusion of CX3CL1-12ALA and of this mutated chemokine without its extracellular domain (“cyto-TM-12ALA”) or without its extra and intracellular domains (“TM-12ALA”) is also reported. *** p<0.001 compared with native CX3CL1-EYFP.

We also assayed the effect of the deletion of the different parts of CX3CL1 on its diffusion rate in the COS7 cell line (Fig. 4A). The truncation of the CD (“w/o CD CX3CL1”) had no effect on the lateral diffusion of the molecule, whereas the truncation of the cytosolic tail (“w/o cyto CX3CL1”) resulted only in a modest 1.2-fold increase in the diffusion rate. In contrast, the truncation of the extracellular region (“cyto-TM”) yielded a net 2-fold increase. Moreover, the truncation of both the intracellular and extracellular regions of CX3CL1 without changing the TM domain resulted in a molecule that was almost 3-fold more mobile than the whole molecule. Thus, it appears that the extracellular part of the chemokine is largely involved in the restraining of its diffusion rate.

To assess the impact of glycosylation on the lateral diffusion of CX3CL1, we used FRAP to test the diffusion of the dgCX3CL1 variant and found that its diffusion rate was more than 2-fold larger than that of native CX3CL1 (Fig. 4A). To confirm that this dramatic increase in the diffusion rate is truly due to the absence of glycosylation, we performed experiments with the glycosylation-defective CHO cell line, termed CHO*ldlD* (Krieger et al., 1981) (Fig. 4B). When these cells were deprived of glycosylation substrates (i.e., with 0.1% SVF), the expressed CX3CL1 was indeed of a lower molecular weight (80 kDa instead of 130 kDa, supplementary material Fig. 4C) and diffused at a 2-fold higher rate (Fig. 4B, left grey bar). In addition, the diffusion rate of the dgCX3CL1 chimera and the TM moieties remained unchanged (Fig. 4B), showing that the conditions that lead to a total absence of cell glycosylation did not influence the lateral diffusion of the control membrane proteins. Thus, it appears that the glycosylation of the mucin stalk of CX3CL1 improves the adhesion potency of CX3CL1 by ensuring the accessibility of the CD (Figs 1 and 2) but induces a decreased diffusion rate (Fig. 4A,B), which was a priori contrary to what would be expected for optimized adhesion.

### Monodisperse CX3CL1 bundles are induced by the transmembrane domain

The diffusion rates of the dgCX3CL1 variant, the TM variant, and CX3CL1 in the glycosylation-defective CHO*ldlD* cells (0.08–0.012 µm^2^ s^−1^) (Fig. 4A,B) appeared slow for a protein with a single transmembrane domain because it was of the same order as that of CX3CR1 (Fig. 3D). This slow diffusion could not be due to glycosylation. We previously showed that CX3CL1 is aggregated and that this aggregation – as assessed by BRET/FRET experiments – is mainly controlled by its TM domain (Hermand et al., 2008). We notably showed that the replacements of the 321–325 residues or of the 326–332 residues by ALA (giving the mutants termed CX3CL1-ALA5 and CX3CL1-ALA7, respectively) had no impact on the aggregation (Hermand et al., 2008). Consistently, these mutations had no effect on the diffusion rate of the molecule (Fig. 4D). When all of the 321–332 residues were replaced by ALA, the resulting CX3CL1 (termed “CX3CL1-ALA12”) was previously found to be neither aggregated nor glycosylated (Hermand et al., 2008). Consistently, we found that the CX3CL1-ALA12 diffusion rate (0.13 µm^2^ s^−1^, Fig. 4D) was two-fold higher than the diffusion rate of dgCX3CL1 (0.07 µm^2^ s^−1^; Fig. 4A), confirming that the slow diffusion of deglycosylated CX3CL1 is due to aggregation. Hence, we concluded that CX3CL1 is presented as a monodisperse bundle (supplementary material Fig. S3) on the surface of cells and that its packing is driven by its transmembrane domain.

## DISCUSSION

**Fig. 5.**
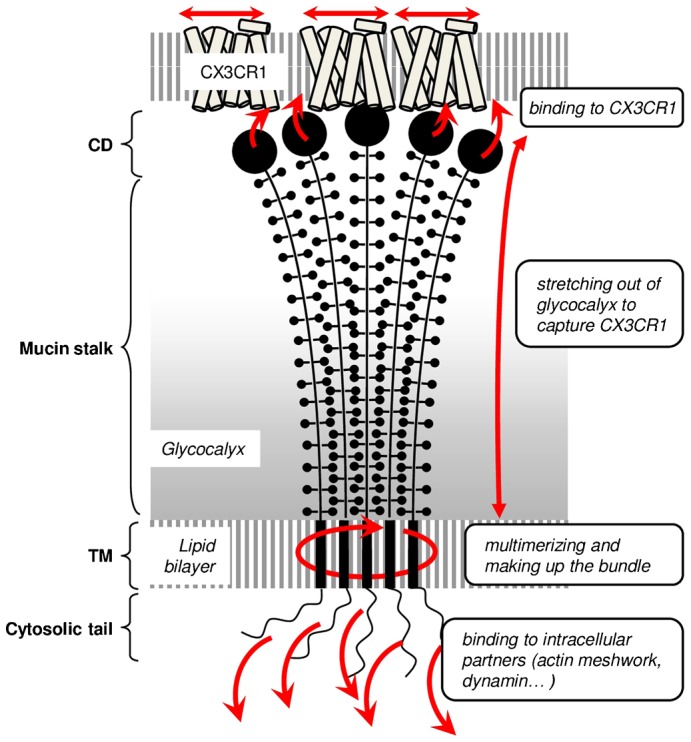
Scheme illustrating the physiological functions of the different CX3CL1 structural domains. The CX3CL1 molecule is quasi-immobile due to the high glycosylation of its mucin stalk and the multimerization driven by its TM domain. The oligomer molecule is catching mobile CX3CR1 receptors through binding to its CD (chemokine domain) thanks to the flexible and stretched mucin stalk. The robustness of the adhesion is ensured by the CX3CL1 cytosolic domain anchoring it to the cytoskeleton.

The adhering chemokine CX3CL1 is natively expressed as a transmembrane molecule consisting of four domains. The CD (76 residues) – a globular protein domain 3 nm in diameter maintained by two disulfide bridges – is structurally similar to that of other secreted chemokines. The mucin stalk (241 residues) is 26 nm in length (Fong et al., 2000) and highly glycosylated with 17 mucin-like repeats (Imai et al., 1997; Fong et al., 1998). The transmembrane domain is involved in the aggregation of the molecule, as assayed by BRET and FRET (Hermand et al., 2008). Finally, the cytosolic domain (C-terminus) is involved in the cellular trafficking of CX3CL1 (Andrzejewski et al., 2010) and in its constitutive endocytosis (Liu et al., 2005; Huang et al., 2009). Using numerous CX3CL1 mutants whose characteristics are summarized in Table 3, our present study provides important clues that aid our understanding of the specific roles of each domain of the CX3CL1 molecule in its adhesive function (Fig. 5).

**Table 3.**
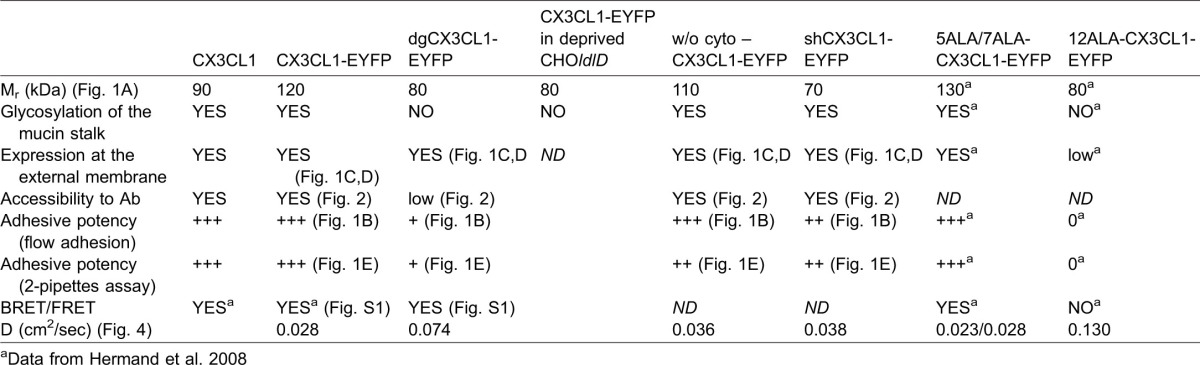
Summary of the data collected about CX3CL1 and its mutants

1. As previously underlined, the CD is the only region of CX3CL1 that binds to the receptor (Fong et al., 1998; Mizoue et al., 2001). Its high affinity (K_d_ of approximately 0.1–1 nM) provides the actual adhesion energy, particularly due to its very low off-rate compared to that of any other chemokine/chemokine receptor pair (Haskell et al., 2000). However, it was recognized that this favorable affinity cannot solely account for the exceptional bond strength afforded by the CX3CL1-CX3CR1 pair when the CX3CL1 molecules are isolated (Lee et al., 2004).

2. Our data provide several lines of evidence supporting the hypothesis that the glycosylated mucin stalk plays an important role in the presentation of the CD to the outer medium. It was previously demonstrated that the presence of the mucin stalk increases the power of the adhesive CX3CL1-CX3CR1 interaction (Fong et al., 2000). The same researchers showed that the E-selectin stalk can replace the mucin domain of CX3CL1 without changing its adhesion potency and proposed that the mucin stalk function is limited to the extension of the chemokine domain away from the endothelial cell surface to present it to flowing leukocytes (Fong et al., 2000). However, the exact role of the glycosylation of the mucin stalk of CX3CL1 was not analyzed. In this study, we demonstrate that the length and the glycosylation state of the stalk domain are directly involved in the adhesive properties of CX3CL1. Two CX3CL1 mutants (see Table 3) presenting either a shorter glycosylated stalk domain or a normal-length but fully deglycosylated stalk domain exhibit a strongly diminished or abolished adhesion ability under flow conditions (Fig. 1B) and under pulling by the dual pipette assay (Fig. 1E). We believed that the reduced adhesion is due to the limited accessibility to the receptor of the CD that is more buried in the glycocalyx. In the case of the shCX3CL1, it is because it cannot extend beyond the glycocalyx. Regarding the dgCX3CL1, the absence of glycosylation reduces the stiffness of the mucin stalk which ends up being not fully extended, explaining why the CD in the dgCX3CL1 variant has less access to its molecular partners, i.e., either the cognate antibody (Fig. 2) or the CX3CR1 receptor, as assessed by the lower probability of adhesion events (Fig. 1E). We cannot formally exclude that the possibility that the mutations of all glycosylation sites in the mucin stalk could generate other structural changes; however our data show that the dgCX3CL1 is targeted to the plasma membrane as well as the native CX3CL1 (Fig. 1C,D) and has the same aggregation status (supplementary material Fig. S1). To bear out that the CD is in a similar conformation in both dgCX3CL1 and native CX3CL1 molecules, we compared its accessibility to specific antibody when the glycocalyx is absent, i.e. in CHO*ldlD* cells in 0.1% SVF condition (supplementary material Fig. S4). We found that the dgCX3CL1 is similarly targeted to the membrane (supplementary material Fig. S4A) and get similar access to the antibody (PE-stained) (supplementary material Fig. S4B). Taken together, our data show that the glycosylation of the CX3CL1 mucin domain is crucial for its stretch out of the profound layer of glycocalyx and for allowing the chemokine domain to obtain access to the receptor on the counter cell, as shown for P-selectin (Patel et al., 1995) and Natural Killer cell receptor NKp30 (Hartmann et al., 2012). This extended CX3CL1 may also allow better interactions with CX3CR1 because a greater intermembrane distance minimizes the repulsive forces between the glycocalyces.

3. As reported for the VSV-G-protein (Scullion et al., 1987) and class I MHC glycoprotein (Wier and Edidin, 1988), we found that the CX3CL1 glycosylation markedly restrains its lateral diffusion, as determined using a deglycosylated CX3CL1 mutant (dgCX3CL1, Fig. 4A) and CX3CL1 expression in the CHO*ldlD* cell line, which is unable to glycosylate under serum-deprived conditions (Fig. 4B).

4. Our study also provides evidence that the transmembrane domain, in addition to anchoring the protein to the membrane, aggregates itself to form a bundle with several CX3CL1. This phenomenon is exemplified by the dramatic difference between the lateral diffusion of the two deglycosylated CX3CL1 variants (see Table 3): the diffusion of CX3CL1-12ALA, which was previously shown to be disaggregated (Hermand et al., 2008) (0.130±0.004 µm^2^ s^−1^, Fig. 4D), is noticeably greater than that of dgCX3CL1 (0.074±0.001 µm^2^ s^−1^, Fig. 4A), which displays the same positive BRET as natural CX3CL1 (supplementary material Fig. S1). Moreover, we found that the diffusion of the TM of the CX3CL1-12ALA mutant (0.177±0.006 µm^2^ s^−1^, Fig. 4D) is more than 2-fold faster than that of the TM of natural CX3CL1 (0.081±0.001 µm^2^ s^−1^, Fig. 4A).

It should be noticed that the BRET data indicate the aggregation status of the CX3CL1 molecule (Hermand et al., 2008) but do not provide any clues regarding the fraction of aggregated molecules or the degree of aggregation. From our FRAP data (Figs 3 and 4), we can infer that this aggregation involves the great majority of the molecules, that it is unchanged even in cells with a low number of expressed CX3CL1 (Fig. 3B) and that the aggregation appears to be relatively homogeneous because the population of mobile CX3CL1 appears to be “monodisperse” (supplementary material Fig. S3). However, more work is required to precisely determine the number of monomers in each CX3CL1 aggregate, even though our data argue that a fixed number of monomers are found in each CX3CL1 bundle, giving rise to a fixed adhesive strength. Finally, our work notes that this aggregation with a fixed number of elements is likely an intrinsic multimerization because it occurs in many cell type environments (Fig. 3 and Table 1).

5. Our data indicate that the cytosolic domain ensures the robustness of the adhesion, probably by anchoring the bundle to some intracellular structures as cytoskeleton. The role of this domain is clearly visible using the dual pipette assay (Fig. 2) and could be due to the interaction of the CX3CL1 cytosolic tail with the actin network, as previously shown for E-cadherin/catenins and integrins adhesion (van Kooyk and Figdor, 2000; Noda et al., 2010). This is also consistent with the decreased adhesion observed in our experiment using latrunculin B (supplementary material Fig. S2). More work is still needed to assert this link. In any case, the truncation of this tail does not affect flow adhesion under our shear stress conditions (Fig. 1B). This indicates that the main resistance to pulling the cell expressing CX3CL1 from the one expressing CX3CR1 comes from the whole CX3CL1 molecule structure and only in a minor part from its links to intracellular partners. This result is in accordance with previous results that demonstrates that the intracellular domain of murine CX3CL1 is not involved in static adhesion (Andrzejewski et al., 2010). This contribution of the cytosolic tail to the adhesive potency of CX3CL1 adds to its role in the recycling of the chemokine by binding to dynamin in order to facilitate constitutive endocytosis (Huang et al., 2009).

Overall, the findings suggest a structural model of the CX3CL1 molecule that appears finely adapted to its adhesive function (Fig. 5): several CX3CL1 molecules with CDs that are perfectly accessible are presented together to circulating leukocytes, and their subsequent adhesion will be robust. The slow diffusion of CX3CL1 may appear to be unfavorable for adhesion. However, because several CX3CL1 molecules are presented together, there is likely no need to bring more CDs to the adhesion area. It is even possible that this slow diffusion guarantees the involvement of a relatively accurate and constant number of CDs, making the initial adhesion more reproducible. The diffusion of CX3CR1 molecules that have to migrate toward the CX3CL1 bundle will control the dynamics of adhesion patch formation. Thus, the comparison of the CX3CL1-CX3CR1 pair to the integrin-integrin ligand couple revealed that it is the receptor rather than the chemokine that behaves as an integrin: its diffusion and its aggregation are directly involved in its interaction with the protruded immobile and multivalent ligand to form an adhesive complex.

CX3CL1 is a special adhesive chemokine that is able to develop a strong specific interaction with its receptor CX3CR1 and is able to catch a circulating monocyte without a rolling step (Haskell et al., 1999). However, this adhesive potency remains lower than that of integrin (Kerfoot et al., 2003). The CX3CL1-CX3CR1 pair also allows the patrolling or “crawling” behavior of the CX3CR1-positive monocytes in the lumen of blood vessels (Auffray et al., 2007) and within the bone marrow (Jacquelin et al., 2013). All of these *in vivo* functions require a finely tuned adhesive potency that perfectly matches the model detailed in the present structural study. For instance, the presentation of CX3CL1 as quasi-immobile bundles has advantages that can explain the patrolling role of the CX3CL1-CX3CL1 pair. To ensure strong binding, the initial CX3CR1-CX3CL1 bond must be followed by the formation of more bonds before it breaks. The lifetime of a single bond is roughly given by τ_0_exp(−ΔG/k_B_T), where the prefactor τ_0_ is ∼10^−8^ s for molecules of this size (Evans, 2001), k_B_T is the thermal energy, and ΔG is the energy of the bond. For K_d_ = 1 nM, ΔG = 20 k_B_T, which results in a lifetime of approximately 5 s. Because the diameter of a monocyte is 8–14 µm (Geissmann et al., 2003; Tacke and Randolph, 2006) and assuming that 2×10^4^ CX3CR1 molecules are found per monocyte (Moatti et al., 2001), the average surface per receptor is approximately 0.002–0.01 µm^2^/CX3CR1. Hence, considering the diffusion coefficient of CX3CR1 (0.1 µm^2^/s), a second bond will form within 0.1 s, i.e., before the first bond dissociates. This finding strongly suggests that because several CX3CL1 molecules are presented together at the same location, the formation of the first bond induces all of the other CX3CL1 molecules of the bundle to be bound to CX3CR1 partners. This process guarantees a strong binding of the monocytes and prevents rolling. However, monocytes must not be permanently attached. Therefore, the total number of CX3CR1-CX3CL1 bonds must remain limited to avoid irreversible adhesion. The slow diffusion of CX3CL1 (either immobile or 10-fold slower than that of CX3CR1) is also an advantage because it likely prevents the formation of too many bonds, thereby making the adhesion reversible and allowing specific functions, such as patrolling.

## MATERIALS AND METHODS

### CX3CL1 constructs

The *cx3cl1* constructs in pcDNA3 (Invitrogen), pEYFP-N1 (Clontech), and pRluc-N2 (Perkin-Elmer) were obtained through PCR amplification using *cx3cl1*-pBLAST (Invivogen Cayla, Toulouse, France) as the template and primers containing a HindIII restriction site in the 5-position and a BamHI restriction site in the 3-position, as described previously (Hermand et al., 2008). The HindIII/BamHI fragment was then cloned into the different plasmids. The truncated and mutated constructs were generated using the QuikChange II site-directed mutagenesis kit according to the manufacturer's instructions (Stratagene). Briefly, 20 ng of the various plasmid constructs was used as the template with mutated nucleotide primers, as described previously (Hermand et al., 2008). The PCR conditions were as follows: predenaturing at 95°C for 1 min followed by 18 cycles of denaturing at 95°C for 50 s, annealing at 60°C for 50 s, and extension at 68°C for 1 min/kb. After digestion with DpnI, 2 µl of the PCR product was used to transform the XL10-Gold ultracompetent cells provided with the kit. The appropriate clones were identified by sequencing.

The oligonucleotide primers used for the mutagenesis of human CX3CL1 are the following. To generate the CX3CL1 without the cytosolic domain from CX3CL1 (w/o cyto CX3CL1), we used 5′-GTGGCCATGTTCACCTACGGGGGAGGGGATCCACCG-3′ and 5′-CGGTGGATCCCCTCCCCCGTAGGTGAACATGGCCAC-3′. To generate the short CX3CL1 (shCX3CL1) from CX3CL1, we used 5′-CCACTGCCGCCACGTGGCAGGTGGGGCTGCTGGCCTTCC-3′ and 5′-GGAAGGCCAGCAGCCCCACCTGCCACGTGGCGGCAGTGG-3′. To generate the deglycosylated CX3CL1 (dgCX3CL1), we used a synthetic gene from GeneArt (Life Technologies, Saint-Aubin, France). The CX3CL1 constructs without the CD (w/o CD CX3CL1), without the mucin stalk (cyto-TM), and with only the transmembrane domain (TM) were performed as previously described (Hermand et al., 2008).

### Cell culture and transfection

A human embryonic kidney cell line (HEK293), a fibroblast-like cell line derived from monkey kidney tissue (COS7), and the Chinese Hamster Ovary cell line (CHO) were grown in DMEM medium supplemented with 10% fetal calf serum (FCS), 1% sodium pyruvate, and antibiotics. The monocytic cell line THP-1 was grown in RPMI medium supplemented with 10% fetal calf serum (FCS), 1% sodium pyruvate, and antibiotics. UDP-Gal/UDP-GalNAc 4-epimerase = deficient mutant Chinese Hamster Ovary (CHO) cells denoted CHO*ldlD* (Kingsley et al., 1986) were grown in Ham F-12 medium supplemented with 10% or 0.1% FCS and antibiotics. The 4-epimerase deficiency prevents the synthesis of UDP-Gal and UDP-GalNAc under normal culture conditions when glucose is the sole sugar source. Transient or stable transfections were performed using JetPei (PolyPlus Transfection, Illkrich, France) according to the manufacturer's instructions. Stably transfected cells were selected with 1 mg/ml geneticin (G418, Life Technologies), and single clones were established by limited dilution. Human umbilical vein endothelial cells (HUVEC) were purchased from Lonza (Basel, Switzerland) and cultured in EBM-2 medium (Lonza) following the manufacturer's instructions. The cells were subcultured before reaching confluence for a maximum of three passages. CX3CL1 expression was induced by the addition of IFN-γ and TNF-α (500 units/ml and 20 ng/ml, respectively), as previously described (Hermand et al., 2008).

### Western blot experiments

Protein samples (30 µg) from cell lysates were loaded on a NuPAGE® Novex® 10% gel (Life Technologies) under reducing conditions, transferred to a nitrocellulose membrane and incubated overnight at 4°C under agitation with anti-human CX3CL1 primary antibody, which recognizes the CD domain (AF365, 1/500 dilution, R&D Systems Europe, Lille, France). Immune complexes were visualized with secondary peroxidase-conjugated antibodies, using a chemiluminescent kit (GE Healthcare Europe, Saclay, France).

### Fluorescence recovery after photobleaching (FRAP) experiments

Cells were seeded into four wells borosilicate slide Labtek II chamber (Nunc, Dutscher, Brumath, France) 48 h before the experiments and transfected (HEK293, COS7, and CHO*ldlD*) or stimulated (HUVEC) 24 h before the FRAP experiments. Stimulated HUVEC cells expressing CX3CL1 were incubated with fluorescein-labeled anti-CX3CL1 polyclonal antibody (IC365F from R&D Systems) 1 h before the experiments. Confocal imaging was performed on live cells with a Leica SP5 microscope using a 488-nm laser beam for EYFP or FITC excitation and the filter sets supplied by the manufacturer. The cells were maintained at 37°C on the microscope stage. In the FRAP experiments, the measurements were performed using a Leica 63× dry objective. Two identical regions of interest (ROI) were monitored: one was photobleached during three scans with the 488 nm laser beam at full power, and the other was used to monitor the concomitant effects of intrinsic photobleaching. The pre- and post-bleach images were monitored at low laser intensity (10 to 15% of full power). The fluorescence in the ROIs was quantified using the LASAF Leica software. The analysis of the curve of the resulting fluorescence recovery as a function of time yielded the recovery times that were used to obtain the diffusion coefficients of the diffusing species. The diffusion coefficient is equal to
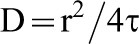
where “r” is the radius of the circular beam and “τ” is the time constant obtained from the fit of the curve (Soumpasis, 1983). In our experiments, each cell was bleached over three different circular regions with diameters of 3, 4, or 5 µm. The characteristic recovery times (τ) and fluorescence recovery plateau (F_p_) were calculated by fitting the fluorescence recovery curves as previously described (Soumpasis, 1983; Braeckmans et al., 2003). The mobile fraction was calculated as the ratio of F_p_ to the initial fluorescence level (F_0_).

### Evaluation of membrane expression of EYFP chimera proteins by confocal microscopy

The membrane expression of different EYFP-CX3CL1 chimeras was evaluated as previously described (Janecki et al., 2000). Briefly, the intensity of EYFP fluorescence corresponding to the plasma membrane was calculated by subtracting the value of the integrated fluorescence intensity of EYFP within the cytoplasm from the total cellular fluorescence intensity over at least five optical sections per cell. We reported the mean of six cells for each condition.

### Flow cytometry

The expression of different EYFP-CX3CL1 chimeras was analyzed by flow cytometry using a FACSCalibur (Becton Dickinson, Le Pont de Claix, France) and the FlowJo software (TreeStar, Ashland, OR, USA). Transiently transfected COS-7 or CHO*ldlD* cells were harvested from culture flasks through treatment with cell dissociation buffer (Life Technologies, Saint Aubin, France), washed in PBS, and centrifuged. The cells were fixed in 0.5 mL of 4% paraformaldehyde in PBS for 10 min on ice, washed with PBS/0.01% NaN_3_, and incubated at a density of 2.10^6^ cells per mL with a phycoerythrin (PE)-conjugated anti-hCX3CL1 monoclonal antibody (R&D Systems) or a PE-conjugated IgG1 isotype control (both at a concentration of 1.25 µg/mL Ab in PBS with 0.1% BSA and 0.01% NaN_3_) for 1 h on ice. After two washes, the cells were suspended in PBS containing 2% PFA.

### Flow adhesion experiments

The laminar flow chamber adhesion assays were performed as previously described (Daoudi et al., 2004). Briefly, Thermanox coverslips (Nunc, Dutscher, Brumath, France) were cultured with adherent COS7 cells transiently expressing the CX3CL1-EYFP, dgCX3CL1-EYFP, CX3CL1-EYFP w/o the cytosolic domain (w/o cyto CX3CL1), or CX3CL1-EYFP w/o the chemokine domain (w/o CD CX3CL1) mutants. The coverslip was mounted in a chamber set on the stage of an inverted microscope (TE300, Nikon) equipped with a phase-contrast 10 objective (Nikon, n.a. 0.25) and a cooled charge-coupled device camera (Sensicam, PCO, Kelheim, Germany). The entire apparatus was maintained at 37°C by a thermostatic chamber (Life Imaging Services, Reinach, Switzerland). CHO-CX3CR1 clone cells were suspended in PBS, incubated for 30 min at 37°C with 5 µM CellTracker™ Orange CMTMR (Molecular Probes, Life Technologies, CA, USA) for labeling, and resuspended in flow buffer (HBSS supplemented with 1 mM CaCl_2_, 1 mM MgCl_2_, 10 mM HEPES, and 2 mg/ml bovine serum albumin) at a density of 10^6^ cells/ml. A syringe pump (PHD 2000; Harvard Apparatus, Les Ulis, France) was used to drive 0.5 mL of the cell suspension through the chamber at a wall shear stress of 1.5 dynes cm^−2^. After a 10-min wash at 1.5 dynes cm^−2^, fluorescent images of six separate 0.5-mm^2^ fields were recorded to count the adherent cells (excitation 450–500 nm, emission 510–560 nm, dichroic filter Q505lp, Chroma, Brattleboro, VT, USA). The number of CHO-CX3CR1 clone cells adhered to COS7 cells expressing CX3CL1 w/o the chemokine domain was considered non-specific adhesion.

### Cell-cell adhesion experiments using the dual pipette aspiration technique

The dissociation force between the stable CX3CR1 clone and CHO and COS7 cells transiently transfected with CX3CL1 was measured through the dual pipette adhesion assay as previously described (Daoudi et al., 2004). The assays were performed on the stage of a Leica inverted microscope, which was positioned on an anti-vibration platform with a digitally controlled thermostat and equipped with 10× and 63× objectives. The incubation chamber consisted of the bottom of a 90-mm Petri dish covered with the inverted bottom of a second dish of the same size. All of the surfaces in contact with the cells were precoated with BSA (5–10% in deionized water) for at least 30 min. To obtain glass pipettes with an inner diameter from 3 to 4 µm, we pulled (with a Sutter instrument, model P-2000), cut, and then fire-polished micropipettes with a homemade microforge. Before the adhesion assay, the pipettes were filled with sterile culture medium and preincubated in BSA. The cells were manipulated with two micropipettes, each of which was held by its own micromanipulator and connected to a combined hydraulic/pneumatic system that provided the necessary control of the aspiration force applied to the cells.

The protocol used in this study was adapted from the method described by Chien and co-workers (Sung et al., 1986). Two cells, which were collected by gentle aspiration onto the tip of each pipette (cell number 1 in pipette A, cell 2 in pipette B), were brought into contact through the use of the micromanipulators and allowed to remain in contact for 5 min. To separate the cells, the aspiration in pipette B was maintained at a level that was sufficiently high to hold cell number 2 tightly, whereas the aspiration in pipette A was increased in a stepwise manner, as measured with a pressure sensor (Validyne: model DP103–38; ranging from 0 to 50,000 Pascal units). After each step, the pipettes were moved apart in an effort to detach the adherent cells from one another. A pair pulled intact from pipette A was moved back to the pipette orifice, the aspiration in the pipette was increased, and another attempt was made to detach the cells from each other. The cycle was repeated until the level of aspiration in pipette A was sufficient to pull one cell from the other. The aspiration employed in each cycle was monitored continuously. In most cases, the cell deformation and contact area variation during the separation process were very limited (less than 20% for the contact area), and the separation took place suddenly, in less than a tenth of a second. The separation force (*F*) for rigid structures can be deduced from the data. The values recorded for each of the last two cycles in the series (*P_n−_*_1_ and *P_n_*) were used to calculate *F* for the pair tested using Eqn 1, where *d* is the internal diameter of pipette A.
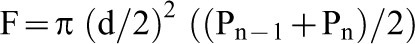
(1)This relation assumes that the pressure inside the cell is the same as that in the chamber, which is valid in our case because the tension of the cell is essentially zero.

### Statistical analysis

The results are expressed as the means ± s.d. from at least 10 measurements. Ana analysis of variance (ANOVA) followed by Tukey test was used to establish the levels of significance.

## Supplementary Material

Supplementary Material
