# Supplementary material for: CX3CL1, a chemokine finely tuned to adhesion: critical roles of the stalk glycosylation and the membrane domain
Source: Biol Open. 2014 Nov 13;3(12):1173–82. doi: 10.1242/bio.20149845 (PMC4265755; doi:10.1242/bio.20149845)
Supplement: Supplementary Material [file supp_3_12_1173__index.html]

CX3CL1, a chemokine finely tuned to adhesion: critical roles of the stalk glycosylation and the membrane domain — CX3CL1, a chemokine finely tuned to adhesion: critical roles of the stalk glycosylation and the membrane domain — Supplementary Material 

# CX3CL1, a chemokine finely tuned to adhesion: critical roles of the stalk glycosylation and the membrane domain

## bio.20149845 Supplementary Material

**Files in this Data Supplement:**

- Supplementary Material - Mariano A. Ostuni et al. doi: 10.1242/bio.20149845
