# Supplementary material for: CX3CL1, a chemokine finely tuned to adhesion: critical roles of the stalk glycosylation and the membrane domain
Source: Biol Open. 2014 Nov 13;3(12):1173–82. doi: 10.1242/bio.20149845 (PMC4265755; doi:10.1242/bio.20149845)
Supplement: Supplementary Material [file supp_bio.20149845_bio.20149845-s1.pdf]

Supplementary Material

Mariano A. Ostuni et al. doi: 10.1242/bio.20149845

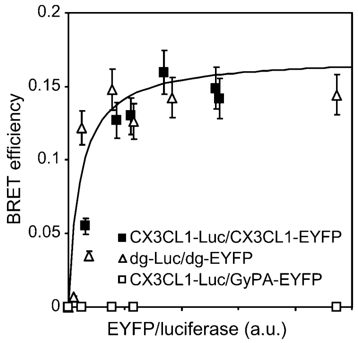

Fig. S1. dgCX3CL1 aggregation determined by BRET. COS7 cells were transiently transfected with luciferase-coupled and EYFP-coupled chimeras of native CX3CL1 or dgCX3CL1 at different EYFP/luciferase ratios. The control of unspecific BRET was performed with COS7 cells transiently transfected with EYFP-coupled glycophorin A (GyPA-EYFP) and luciferase-coupled CX3CL1. The BRET experiments were performed as previously described (Hermand et al., 2008).

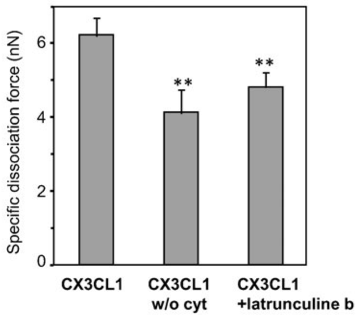

Fig. S2. Role of cytosolic domain in adhesion force. Dissociation force between CX3CR1 stable CHO clone and COS7 cells transiently transfected with CX3CL1-EYFP or with CX3CL1-EYFP w/o cyt. The cells expressing CX3CL1-EYFP were pretreated or not with 500 nM Latrunculin B for 60 min before the experiments. ** $p < 0.01$ compared with native CX3CL1-EYFP.

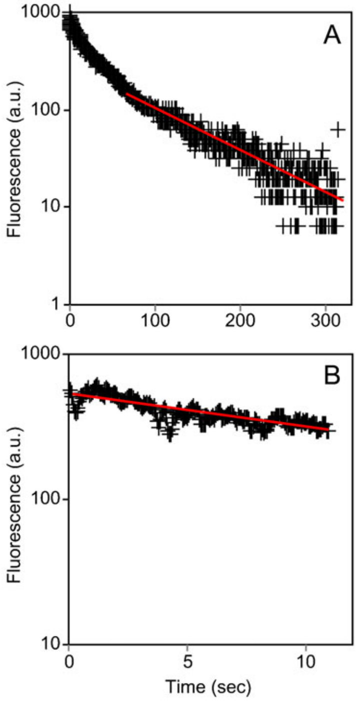

Fig. S3. Monodisperse character of the fluorescence recovery of CX3CL1-EYFP in COS cells as analyzed by FRAPP. (A) Typical fluorescence recovery curve by FRAPP using an interfringe of 4.4 μm. The Y-axis shows the difference in intensity between the bright and dark fringes, F(t). This difference reaches zero when the fluorescence is fully recovered. The long time scale behaves as a single exponential and corresponds to the recovery of the slow CX3CL1 population. The exponential fit ($F_{slow}(t)$) gives a characteristic time corresponding to a diffusion coefficient of $D_{slow}=0.005\ \mu m^2/s$. (B) Plot of the contribution of the short timescale behavior $F(t)-F_{slow}(t)$, which also appears to be a single exponential with a characteristic time that corresponds to a diffusion coefficient of $D_{fast}=0.025\ \mu m^2/s$. In this example, 67% of the CX3CL1 molecules in the population are diffusing in the fast regime. The pure exponential behavior indicates that the diffusive objects (bundles of CX3CL1) are essentially monodisperse in size.

Biology Open (2014) 000, 1–10 doi:10.1242/bio.20149845

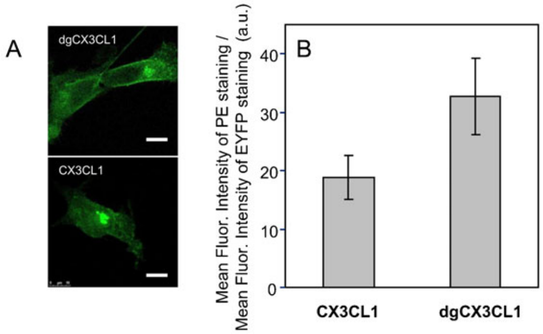

Fig. S4. Membrane localization of CX3CL1 and dgCX3CL1 expressed in CHOldlD cells under low serum conditions (0.1% FCS). (A) Representative images of CHOldlD cell expressing dgCX3CL1 (top panel) and native CX3CL1 (bottom panel). Images were taken with a Leica SP5 confocal microscope (63× dry objective). The bars represent 10 µm. (B) Flow cytometry signal ratio between anti-CX3CL1 PE-conjugated antibody fluorescence and EYFP fluorescence. CHOldlD cells expressing dgCX3CL1-EYFP or native CX3CL1-EYFP were cultured under low serum condition and labelled using anti-CX3CL1 PE-conjugated antibody. Fluorescence signal was analyzed by flow cytometry.

Table S1. Characteristics of the lateral diffusion of CX3CL1 and CX3CR1 in various cell types measured through the FRAPP technique

Cell type	HEK293	HEK293	HEK293 TIRF	HEK293 TIRF
Transfection and fluorescent staining	Stable<br>CX3CL1<br>EYFP	Stable<br>CX3CR1<br>EYFP	Stable<br>CX3CL1<br>EYFP	Stable<br>CX3CR1<br>EYFP
D (μm^2 s^{-1})	0.0178±0.0033	0.0550±0.0076	0.0255±0.0028	0.0549±0.0035

Biology Open
